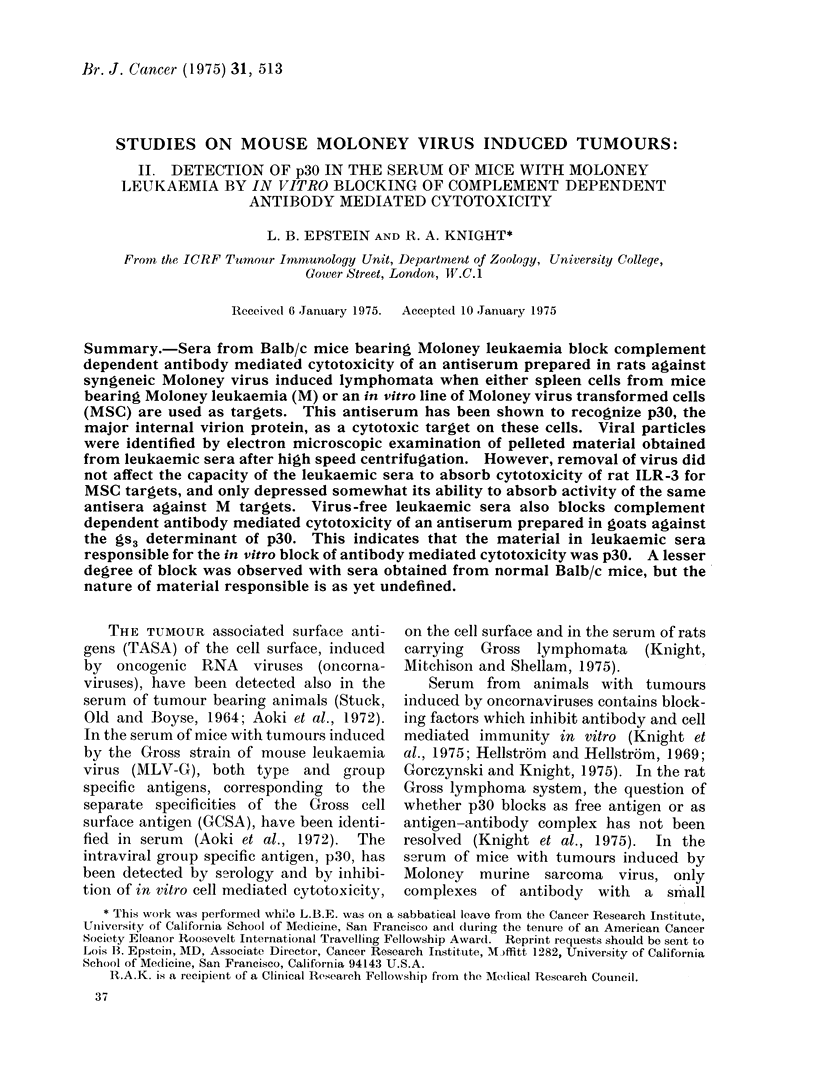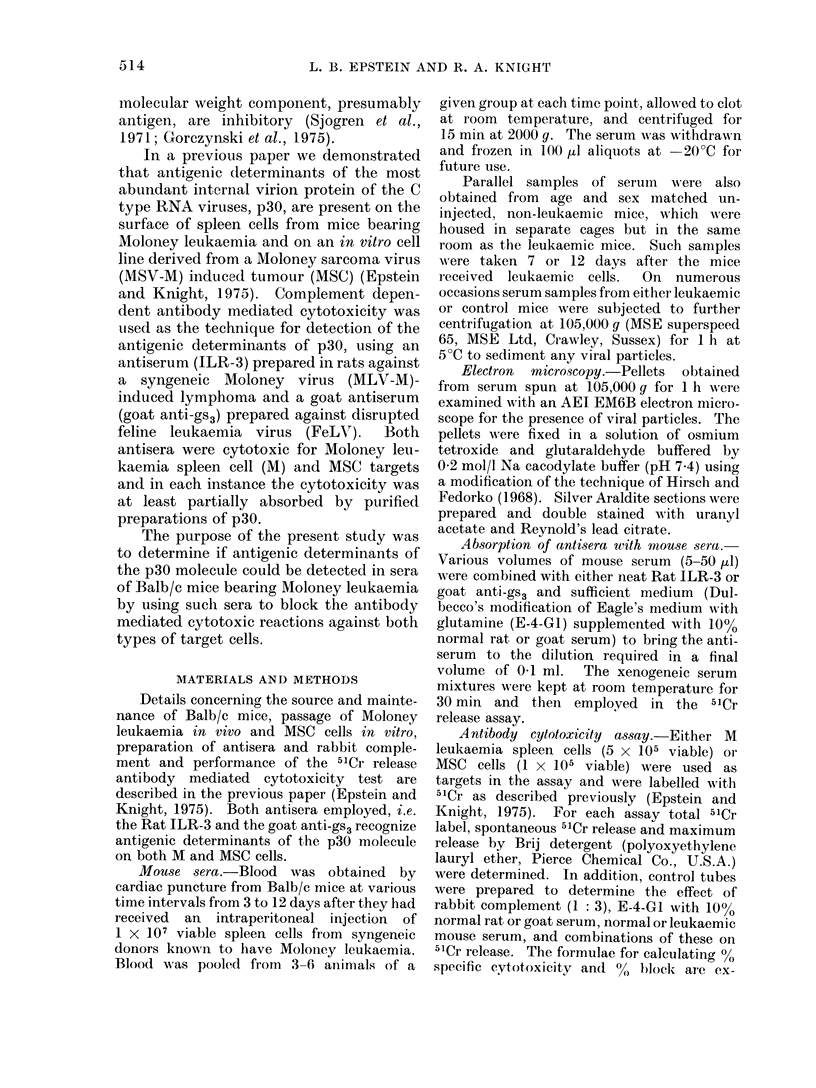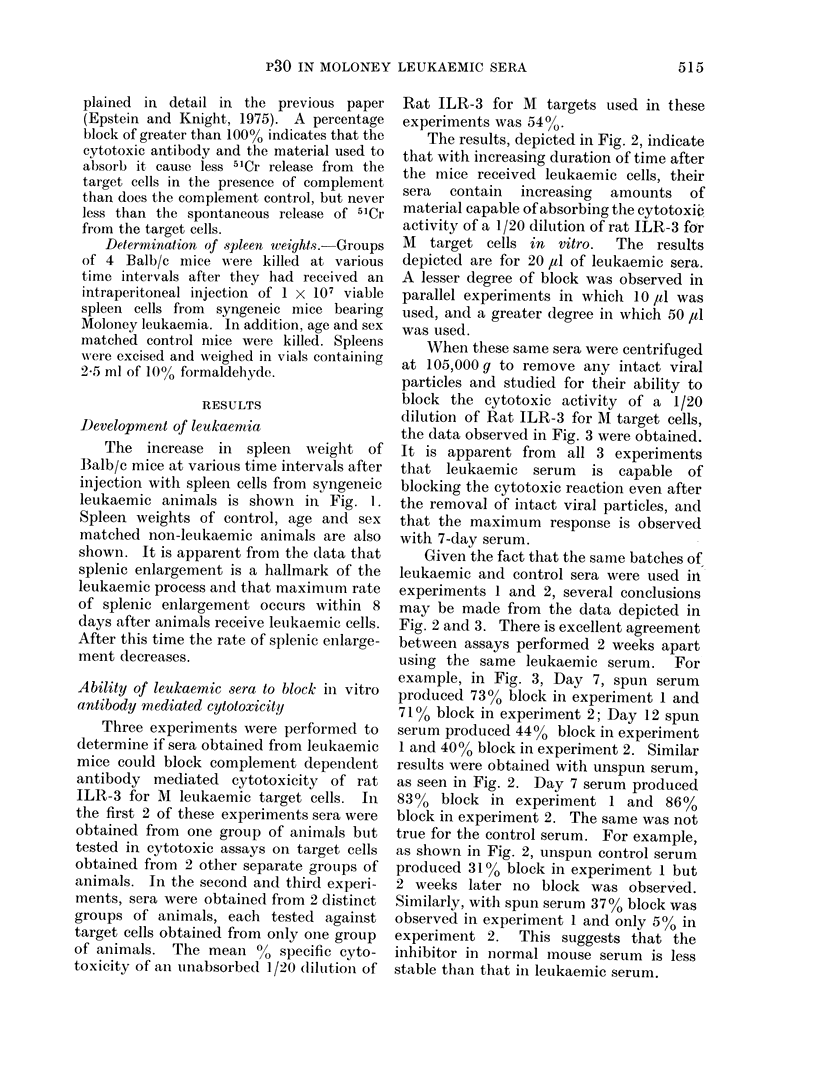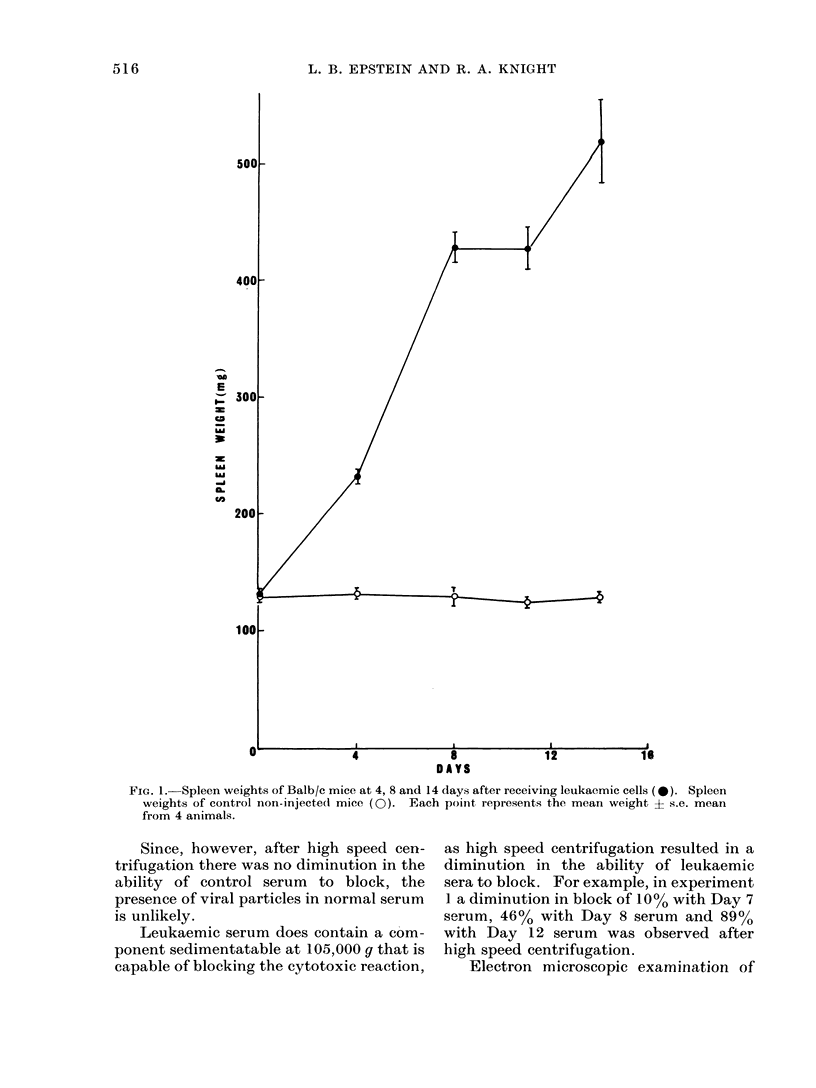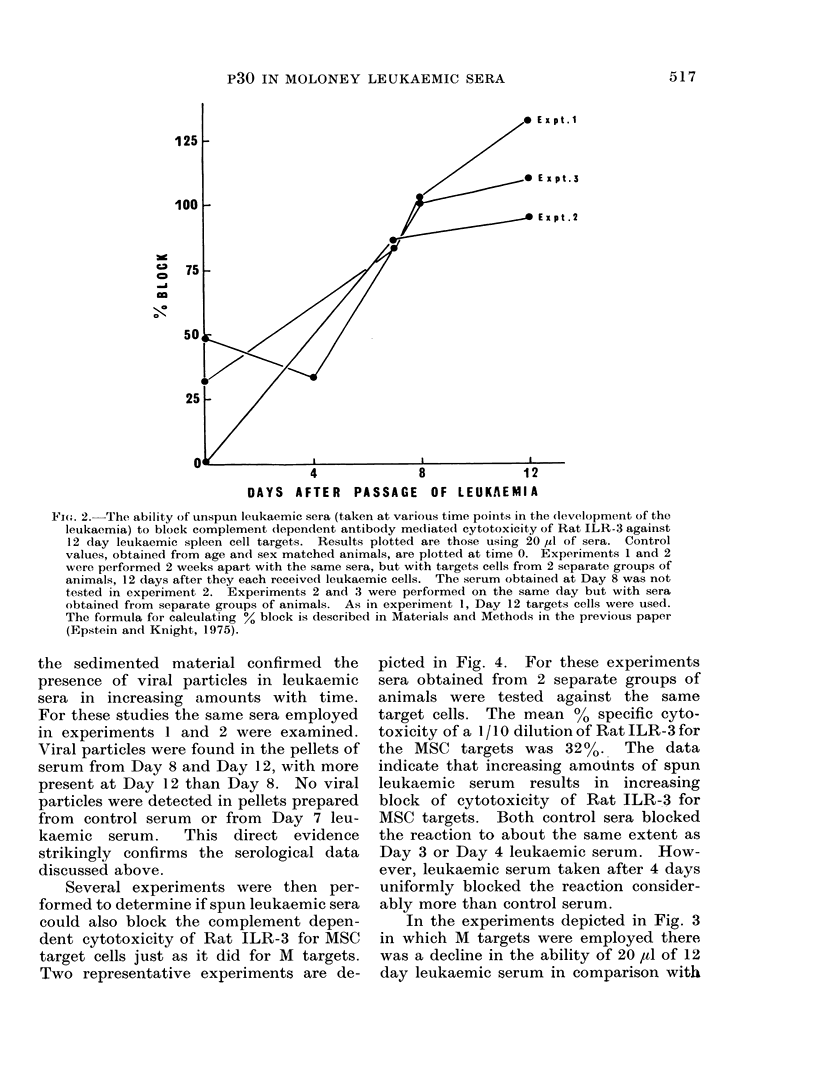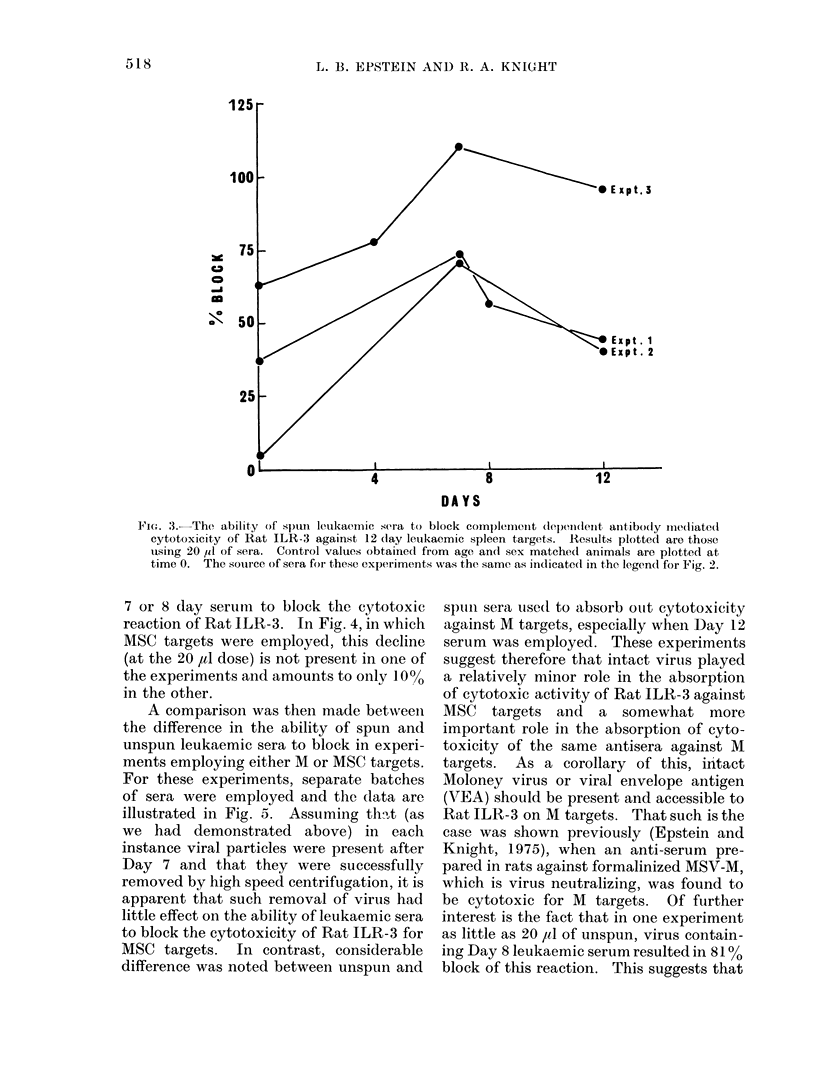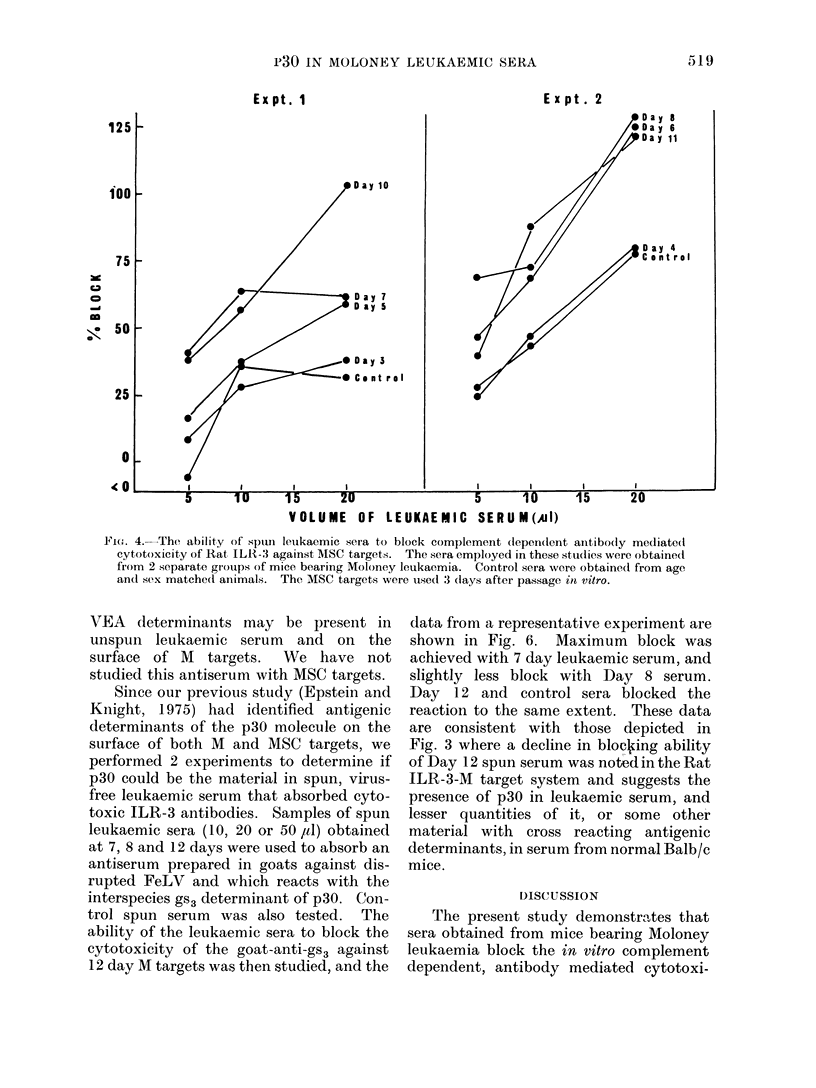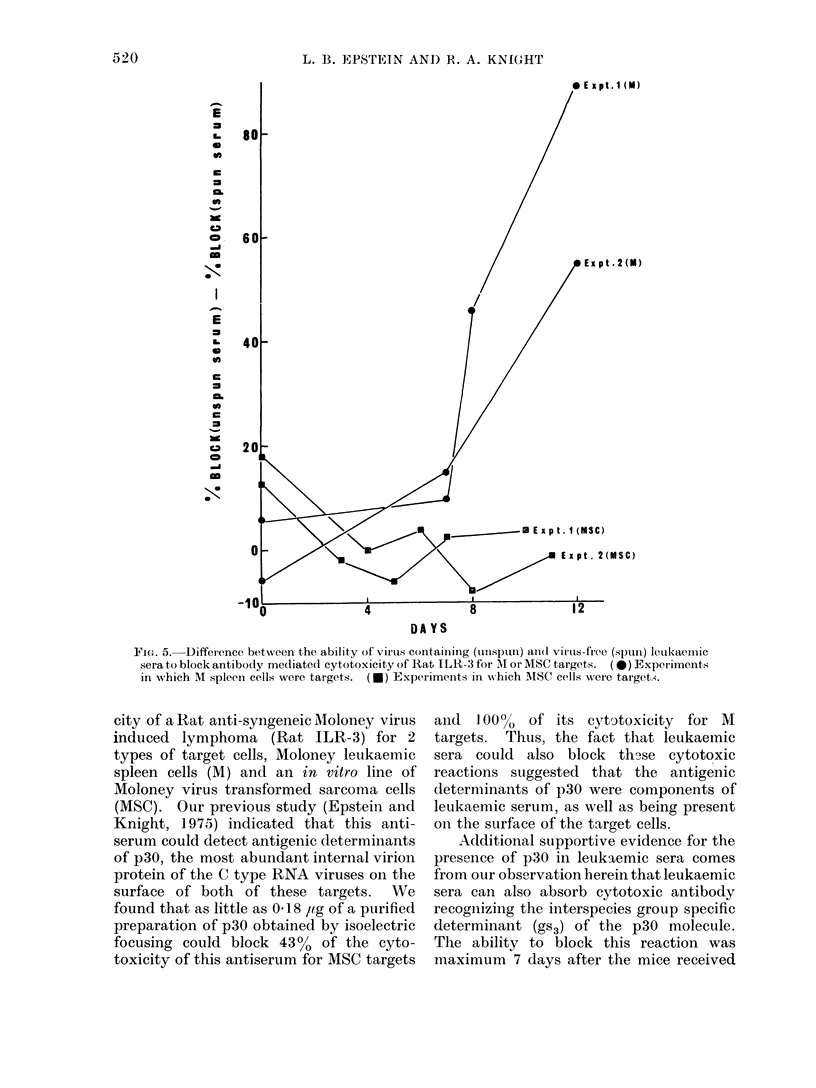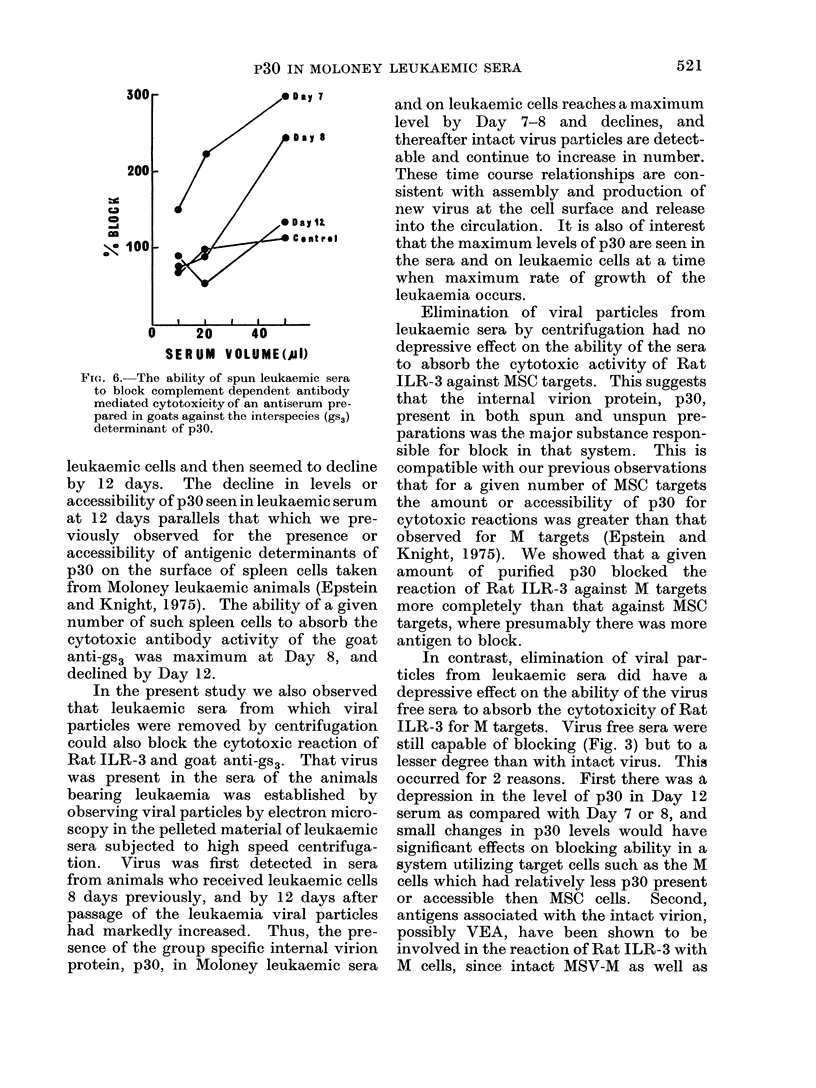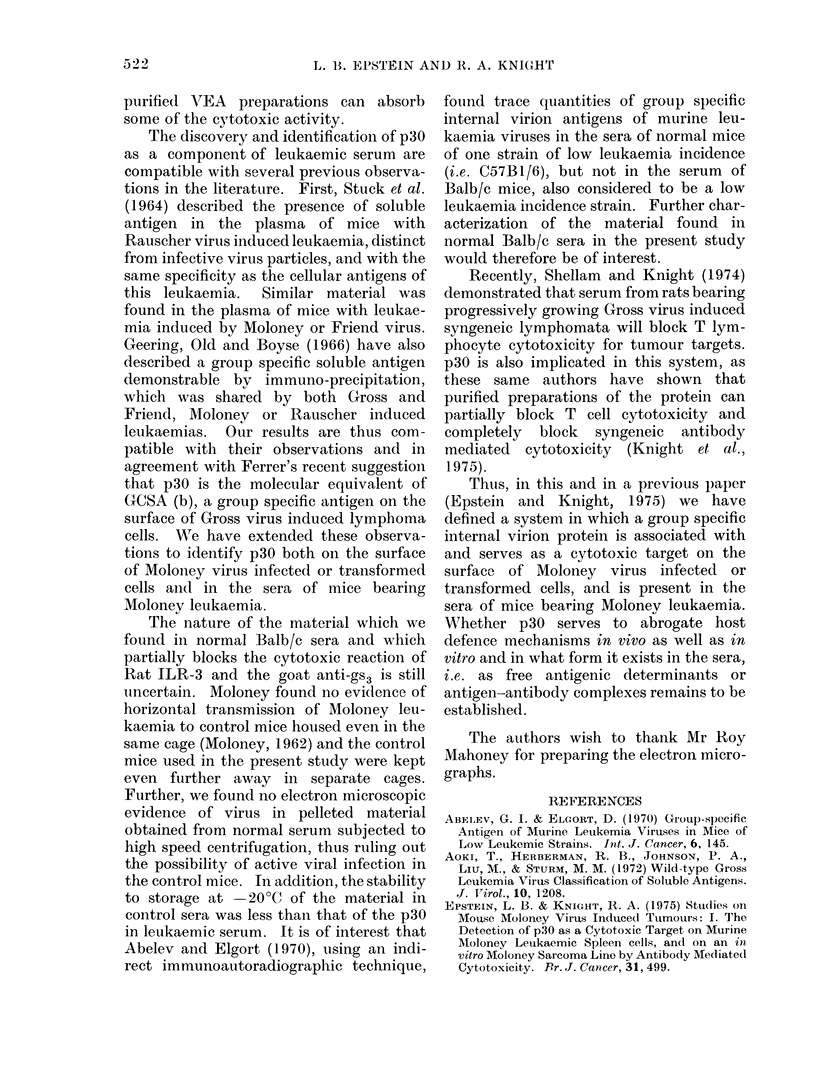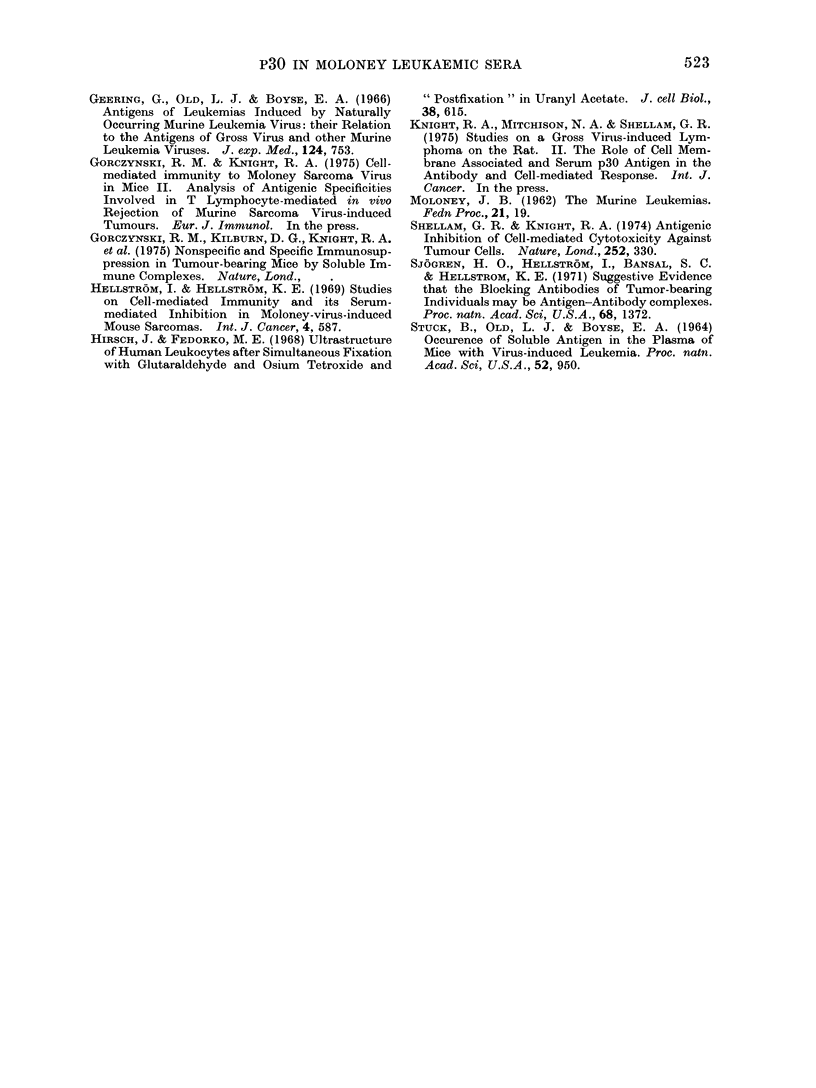# Studies on mouse Moloney virus induced tumours: II. Detection of p30 in the serum of mice with Moloney leukaemia by in vitro blocking of complement dependent antibody mediated cytotoxicity.

**DOI:** 10.1038/bjc.1975.91

**Published:** 1975-05

**Authors:** L. B. Epstein, R. A. Knight

## Abstract

Sera from Balb/c mice bearing Moloney leukaemia block complement dependent antibody mediated cytotoxicity of an antiserum prepared in rats against syngeneic Moloney virus induced lymphomata when either spleen cells from mice bearing Moloney leukaemia (M) or an in vitro line of Moloney virus transformed cells (MSC) are used as targets. This antiserum has been shown to recognize p30, the major internal virion protein, as a cytotoxic target on these cells. Viral particles were identified by electron microscopic examination of pelleted material obtained from leukaemic sera after high speed centrifugation. However, removal of virus did not affect the capacity of the leukaemic sera to absorb cytotoxicity of rat ILR-3 for MSC targets, and only depressed somewhat its ability to absorb activity of the same antisera against M targets. Virus-free leukaemic sera also blocks complement dependent antibody mediated cytotoxicity of an antiserum prepared in goats against the gs3 determinant of p30. This indicates that the material in leukaemic sera responsible for the in vitro block of antibody mediated cytotoxicity was p30. A lesser degree of block was observed with sera obtained from normal Balb/c mice, but the nature of material responsible is as yet undefined.


*Br. J. Cancer* (1975) **31**, 513

### STUDIES ON MOUSE MOLONEY VIRUS INDUCED TUMOURS:

### II. DETECTION OF p30 IN THE SERUM OF MICE WITH MOLONEY LEUKAEMIA BY *IN VITRO* BLOCKING OF COMPLEMENT DEPENDENT ANTIBODY MEDIATED CYTOTOXICITY

L. B. EPSTEIN AND R. A. KNIGHT*

*From the ICRF Tumour Immunology Unit, Department of Zoology, University College, Gower Street, London, W.C.1*

Received 6 January 1975. Accepted 10 January 1975

**Summary.—Sera from Balb/c mice bearing Moloney leukaemia block complement dependent antibody mediated cytotoxicity of an antiserum prepared in rats against syngeneic Moloney virus induced lymphomata when either spleen cells from mice bearing Moloney leukaemia (M) or an *in vitro* line of Moloney virus transformed cells (MSC) are used as targets. This antiserum has been shown to recognize p30, the major internal virion protein, as a cytotoxic target on these cells. Viral particles were identified by electron microscopic examination of pelleted material obtained from leukaemic sera after high speed centrifugation. However, removal of virus did not affect the capacity of the leukaemic sera to absorb cytotoxicity of rat ILR-3 for MSC targets, and only depressed somewhat its ability to absorb activity of the same antisera against M targets. Virus-free leukaemic sera also blocks complement dependent antibody mediated cytotoxicity of an antiserum prepared in goats against the $gs_3$ determinant of p30. This indicates that the material in leukaemic sera responsible for the *in vitro* block of antibody mediated cytotoxicity was p30. A lesser degree of block was observed with sera obtained from normal Balb/c mice, but the nature of material responsible is as yet undefined.**

THE TUMOUR associated surface antigens (TASA) of the cell surface, induced by oncogenic RNA viruses (oncornaviruses), have been detected also in the serum of tumour bearing animals (Stuck, Old and Boyse, 1964; Aoki *et al.*, 1972). In the serum of mice with tumours induced by the Gross strain of mouse leukaemia virus (MLV-G), both type and group specific antigens, corresponding to the separate specificities of the Gross cell surface antigen (GCSA), have been identified in serum (Aoki *et al.*, 1972). The intraviral group specific antigen, p30, has been detected by serology and by inhibition of *in vitro* cell mediated cytotoxicity, on the cell surface and in the serum of rats carrying Gross lymphomata (Knight, Mitchison and Shellam, 1975).

Serum from animals with tumours induced by oncornaviruses contains blocking factors which inhibit antibody and cell mediated immunity *in vitro* (Knight *et al.*, 1975; Hellström and Hellström, 1969; Gorczynski and Knight, 1975). In the rat Gross lymphoma system, the question of whether p30 blocks as free antigen or as antigen–antibody complex has not been resolved (Knight *et al.*, 1975). In the serum of mice with tumours induced by Moloney murine sarcoma virus, only complexes of antibody with a small

* This work was performed while L.B.E. was on a sabbatical leave from the Cancer Research Institute, University of California School of Medicine, San Francisco and during the tenure of an American Cancer Society Eleanor Roosevelt International Travelling Fellowship Award. Reprint requests should be sent to Lois B. Epstein, MD, Associate Director, Cancer Research Institute, Moffitt 1282, University of California School of Medicine, San Francisco, California 94143 U.S.A.

R.A.K. is a recipient of a Clinical Research Fellowship from the Medical Research Council.

molecular weight component, presumably antigen, are inhibitory (Sjogren *et al.*, 1971; Gorczynski *et al.*, 1975).

In a previous paper we demonstrated that antigenic determinants of the most abundant internal virion protein of the C type RNA viruses, p30, are present on the surface of spleen cells from mice bearing Moloney leukaemia and on an *in vitro* cell line derived from a Moloney sarcoma virus (MSV-M) induced tumour (MSC) (Epstein and Knight, 1975). Complement dependent antibody mediated cytotoxicity was used as the technique for detection of the antigenic determinants of p30, using an antiserum (ILR-3) prepared in rats against a syngeneic Moloney virus (MLV-M)-induced lymphoma and a goat antiserum (goat anti-$gs_3$) prepared against disrupted feline leukaemia virus (FeLV). Both antisera were cytotoxic for Moloney leukaemia spleen cell (M) and MSC targets and in each instance the cytotoxicity was at least partially absorbed by purified preparations of p30.

The purpose of the present study was to determine if antigenic determinants of the p30 molecule could be detected in sera of Balb/c mice bearing Moloney leukaemia by using such sera to block the antibody mediated cytotoxic reactions against both types of target cells.

### MATERIALS AND METHODS

Details concerning the source and maintenance of Balb/c mice, passage of Moloney leukaemia *in vivo* and MSC cells *in vitro*, preparation of antisera and rabbit complement and performance of the $^{51}Cr$ release antibody mediated cytotoxicity test are described in the previous paper (Epstein and Knight, 1975). Both antisera employed, *i.e.* the Rat ILR-3 and the goat anti-$gs_3$ recognize antigenic determinants of the p30 molecule on both M and MSC cells.

*Mouse sera.*—Blood was obtained by cardiac puncture from Balb/c mice at various time intervals from 3 to 12 days after they had received an intraperitoneal injection of $1 \times 10^7$ viable spleen cells from syngeneic donors known to have Moloney leukaemia. Blood was pooled from 3–6 animals of a given group at each time point, allowed to clot at room temperature, and centrifuged for 15 min at 2000 $g$. The serum was withdrawn and frozen in 100 $\mu$l aliquots at $-20$°C for future use.

Parallel samples of serum were also obtained from age and sex matched uninjected, non-leukaemic mice, which were housed in separate cages but in the same room as the leukaemic mice. Such samples were taken 7 or 12 days after the mice received leukaemic cells. On numerous occasions serum samples from either leukaemic or control mice were subjected to further centrifugation at 105,000 $g$ (MSE superspeed 65, MSE Ltd, Crawley, Sussex) for 1 h at 5°C to sediment any viral particles.

*Electron microscopy.*—Pellets obtained from serum spun at 105,000 $g$ for 1 h were examined with an AEI EM6B electron microscope for the presence of viral particles. The pellets were fixed in a solution of osmium tetroxide and glutaraldehyde buffered by 0·2 mol/l Na cacodylate buffer (pH 7·4) using a modification of the technique of Hirsch and Fedorko (1968). Silver Araldite sections were prepared and double stained with uranyl acetate and Reynold's lead citrate.

*Absorption of antisera with mouse sera.*—Various volumes of mouse serum (5–50 $\mu$l) were combined with either neat Rat ILR-3 or goat anti-$gs_3$ and sufficient medium (Dulbecco's modification of Eagle's medium with glutamine (E-4-Gl) supplemented with 10% normal rat or goat serum) to bring the antiserum to the dilution required in a final volume of 0·1 ml. The xenogeneic serum mixtures were kept at room temperature for 30 min and then employed in the $^{51}Cr$ release assay.

*Antibody cytotoxicity assay.*—Either M leukaemia spleen cells ($5 \times 10^5$ viable) or MSC cells ($1 \times 10^5$ viable) were used as targets in the assay and were labelled with $^{51}Cr$ as described previously (Epstein and Knight, 1975). For each assay total $^{51}Cr$ label, spontaneous $^{51}Cr$ release and maximum release by Brij detergent (polyoxyethylene lauryl ether, Pierce Chemical Co., U.S.A.) were determined. In addition, control tubes were prepared to determine the effect of rabbit complement (1 : 3), E-4-Gl with 10% normal rat or goat serum, normal or leukaemic mouse serum, and combinations of these on $^{51}Cr$ release. The formulae for calculating % specific cytotoxicity and % block are explained in detail in the previous paper (Epstein and Knight, 1975). A percentage block of greater than 100% indicates that the cytotoxic antibody and the material used to absorb it cause less $^{51}Cr$ release from the target cells in the presence of complement than does the complement control, but never less than the spontaneous release of $^{51}Cr$ from the target cells.

*Determination of spleen weights.*—Groups of 4 Balb/c mice were killed at various time intervals after they had received an intraperitoneal injection of $1 \times 10^7$ viable spleen cells from syngeneic mice bearing Moloney leukaemia. In addition, age and sex matched control mice were killed. Spleens were excised and weighed in vials containing 2·5 ml of 10% formaldehyde.

### RESULTS

### *Development of leukaemia*

The increase in spleen weight of Balb/c mice at various time intervals after injection with spleen cells from syngeneic leukaemic animals is shown in Fig. 1. Spleen weights of control, age and sex matched non-leukaemic animals are also shown. It is apparent from the data that splenic enlargement is a hallmark of the leukaemic process and that maximum rate of splenic enlargement occurs within 8 days after animals receive leukaemic cells. After this time the rate of splenic enlargement decreases.

### *Ability of leukaemic sera to block* in vitro *antibody mediated cytotoxicity*

Three experiments were performed to determine if sera obtained from leukaemic mice could block complement dependent antibody mediated cytotoxicity of rat ILR-3 for M leukaemic target cells. In the first 2 of these experiments sera were obtained from one group of animals but tested in cytotoxic assays on target cells obtained from 2 other separate groups of animals. In the second and third experiments, sera were obtained from 2 distinct groups of animals, each tested against target cells obtained from only one group of animals. The mean % specific cytotoxicity of an unabsorbed 1/20 dilution of Rat ILR-3 for M targets used in these experiments was 54%.

The results, depicted in Fig. 2, indicate that with increasing duration of time after the mice received leukaemic cells, their sera contain increasing amounts of material capable of absorbing the cytotoxic activity of a 1/20 dilution of rat ILR-3 for M target cells *in vitro*. The results depicted are for 20 $\mu$l of leukaemic sera. A lesser degree of block was observed in parallel experiments in which 10 $\mu$l was used, and a greater degree in which 50 $\mu$l was used.

When these same sera were centrifuged at 105,000 $g$ to remove any intact viral particles and studied for their ability to block the cytotoxic activity of a 1/20 dilution of Rat ILR-3 for M target cells, the data observed in Fig. 3 were obtained. It is apparent from all 3 experiments that leukaemic serum is capable of blocking the cytotoxic reaction even after the removal of intact viral particles, and that the maximum response is observed with 7-day serum.

Given the fact that the same batches of leukaemic and control sera were used in experiments 1 and 2, several conclusions may be made from the data depicted in Fig. 2 and 3. There is excellent agreement between assays performed 2 weeks apart using the same leukaemic serum. For example, in Fig. 3, Day 7, spun serum produced 73% block in experiment 1 and 71% block in experiment 2; Day 12 spun serum produced 44% block in experiment 1 and 40% block in experiment 2. Similar results were obtained with unspun serum, as seen in Fig. 2. Day 7 serum produced 83% block in experiment 1 and 86% block in experiment 2. The same was not true for the control serum. For example, as shown in Fig. 2, unspun control serum produced 31% block in experiment 1 but 2 weeks later no block was observed. Similarly, with spun serum 37% block was observed in experiment 1 and only 5% in experiment 2. This suggests that the inhibitor in normal mouse serum is less stable than that in leukaemic serum.

FIG. 1.—Spleen weights of Balb/c mice at 4, 8 and 14 days after receiving leukaemic cells (●). Spleen weights of control non-injected mice (○). Each point represents the mean weight ± s.e. mean from 4 animals.

Since, however, after high speed centrifugation there was no diminution in the ability of control serum to block, the presence of viral particles in normal serum is unlikely.

Leukaemic serum does contain a component sedimentatable at 105,000 *g* that is capable of blocking the cytotoxic reaction, as high speed centrifugation resulted in a diminution in the ability of leukaemic sera to block. For example, in experiment 1 a diminution in block of 10% with Day 7 serum, 46% with Day 8 serum and 89% with Day 12 serum was observed after high speed centrifugation.

Electron microscopic examination of

FIG. 2.—The ability of unspun leukaemic sera (taken at various time points in the development of the leukaemia) to block complement dependent antibody mediated cytotoxicity of Rat ILR-3 against 12 day leukaemic spleen cell targets. Results plotted are those using 20 μl of sera. Control values, obtained from age and sex matched animals, are plotted at time 0. Experiments 1 and 2 were performed 2 weeks apart with the same sera, but with targets cells from 2 separate groups of animals, 12 days after they each received leukaemic cells. The serum obtained at Day 8 was not tested in experiment 2. Experiments 2 and 3 were performed on the same day but with sera obtained from separate groups of animals. As in experiment 1, Day 12 targets cells were used. The formula for calculating % block is described in Materials and Methods in the previous paper (Epstein and Knight, 1975).

the sedimented material confirmed the presence of viral particles in leukaemic sera in increasing amounts with time. For these studies the same sera employed in experiments 1 and 2 were examined. Viral particles were found in the pellets of serum from Day 8 and Day 12, with more present at Day 12 than Day 8. No viral particles were detected in pellets prepared from control serum or from Day 7 leukaemic serum. This direct evidence strikingly confirms the serological data discussed above.

Several experiments were then performed to determine if spun leukaemic sera could also block the complement dependent cytotoxicity of Rat ILR-3 for MSC target cells just as it did for M targets. Two representative experiments are depicted in Fig. 4. For these experiments sera obtained from 2 separate groups of animals were tested against the same target cells. The mean % specific cytotoxicity of a 1/10 dilution of Rat ILR-3 for the MSC targets was 32%. The data indicate that increasing amounts of spun leukaemic serum results in increasing block of cytotoxicity of Rat ILR-3 for MSC targets. Both control sera blocked the reaction to about the same extent as Day 3 or Day 4 leukaemic serum. However, leukaemic serum taken after 4 days uniformly blocked the reaction considerably more than control serum.

In the experiments depicted in Fig. 3 in which M targets were employed there was a decline in the ability of 20 μl of 12 day leukaemic serum in comparison with

FIG. 3.—The ability of spun leukaemic sera to block complement dependent antibody mediated cytotoxicity of Rat ILR-3 against 12 day leukaemic spleen targets. Results plotted are those using 20 μl of sera. Control values obtained from age and sex matched animals are plotted at time 0. The source of sera for these experiments was the same as indicated in the legend for Fig. 2.

7 or 8 day serum to block the cytotoxic reaction of Rat ILR-3. In Fig. 4, in which MSC targets were employed, this decline (at the 20 μl dose) is not present in one of the experiments and amounts to only 10% in the other.

A comparison was then made between the difference in the ability of spun and unspun leukaemic sera to block in experiments employing either M or MSC targets. For these experiments, separate batches of sera were employed and the data are illustrated in Fig. 5. Assuming that (as we had demonstrated above) in each instance viral particles were present after Day 7 and that they were successfully removed by high speed centrifugation, it is apparent that such removal of virus had little effect on the ability of leukaemic sera to block the cytotoxicity of Rat ILR-3 for MSC targets. In contrast, considerable difference was noted between unspun and spun sera used to absorb out cytotoxicity against M targets, especially when Day 12 serum was employed. These experiments suggest therefore that intact virus played a relatively minor role in the absorption of cytotoxic activity of Rat ILR-3 against MSC targets and a somewhat more important role in the absorption of cytotoxicity of the same antisera against M targets. As a corollary of this, intact Moloney virus or viral envelope antigen (VEA) should be present and accessible to Rat ILR-3 on M targets. That such is the case was shown previously (Epstein and Knight, 1975), when an anti-serum prepared in rats against formalinized MSV-M, which is virus neutralizing, was found to be cytotoxic for M targets. Of further interest is the fact that in one experiment as little as 20 μl of unspun, virus containing Day 8 leukaemic serum resulted in 81% block of this reaction. This suggests that

FIG. 4.—The ability of spun leukaemic sera to block complement dependent antibody mediated cytotoxicity of Rat ILR-3 against MSC targets. The sera employed in these studies were obtained from 2 separate groups of mice bearing Moloney leukaemia. Control sera were obtained from age and sex matched animals. The MSC targets were used 3 days after passage *in vitro*.

VEA determinants may be present in unspun leukaemic serum and on the surface of M targets. We have not studied this antiserum with MSC targets.

Since our previous study (Epstein and Knight, 1975) had identified antigenic determinants of the p30 molecule on the surface of both M and MSC targets, we performed 2 experiments to determine if p30 could be the material in spun, virus-free leukaemic serum that absorbed cytotoxic ILR-3 antibodies. Samples of spun leukaemic sera (10, 20 or 50 μl) obtained at 7, 8 and 12 days were used to absorb an antiserum prepared in goats against disrupted FeLV and which reacts with the interspecies $gs_3$ determinant of p30. Control spun serum was also tested. The ability of the leukaemic sera to block the cytotoxicity of the goat-anti-$gs_3$ against 12 day M targets was then studied, and the data from a representative experiment are shown in Fig. 6. Maximum block was achieved with 7 day leukaemic serum, and slightly less block with Day 8 serum. Day 12 and control sera blocked the reaction to the same extent. These data are consistent with those depicted in Fig. 3 where a decline in blocking ability of Day 12 spun serum was noted in the Rat ILR-3-M target system and suggests the presence of p30 in leukaemic serum, and lesser quantities of it, or some other material with cross reacting antigenic determinants, in serum from normal Balb/c mice.

### DISCUSSION

The present study demonstrates that sera obtained from mice bearing Moloney leukaemia block the *in vitro* complement dependent, antibody mediated cytotoxi-

FIG. 5.—Difference between the ability of virus containing (unspun) and virus-free (spun) leukaemic sera to block antibody mediated cytotoxicity of Rat ILR-3 for M or MSC targets. (●) Experiments in which M spleen cells were targets. (■) Experiments in which MSC cells were targets.

city of a Rat anti-syngeneic Moloney virus induced lymphoma (Rat ILR-3) for 2 types of target cells, Moloney leukaemic spleen cells (M) and an *in vitro* line of Moloney virus transformed sarcoma cells (MSC). Our previous study (Epstein and Knight, 1975) indicated that this antiserum could detect antigenic determinants of p30, the most abundant internal virion protein of the C type RNA viruses on the surface of both of these targets. We found that as little as 0·18 μg of a purified preparation of p30 obtained by isoelectric focusing could block 43% of the cytotoxicity of this antiserum for MSC targets and 100% of its cytotoxicity for M targets. Thus, the fact that leukaemic sera could also block these cytotoxic reactions suggested that the antigenic determinants of p30 were components of leukaemic serum, as well as being present on the surface of the target cells.

Additional supportive evidence for the presence of p30 in leukaemic sera comes from our observation herein that leukaemic sera can also absorb cytotoxic antibody recognizing the interspecies group specific determinant ($gs_3$) of the p30 molecule. The ability to block this reaction was maximum 7 days after the mice received

FIG. 6.—The ability of spun leukaemic sera to block complement dependent antibody mediated cytotoxicity of an antiserum prepared in goats against the interspecies ($gs_3$) determinant of p30.

leukaemic cells and then seemed to decline by 12 days. The decline in levels or accessibility of p30 seen in leukaemic serum at 12 days parallels that which we previously observed for the presence or accessibility of antigenic determinants of p30 on the surface of spleen cells taken from Moloney leukaemic animals (Epstein and Knight, 1975). The ability of a given number of such spleen cells to absorb the cytotoxic antibody activity of the goat anti-$gs_3$ was maximum at Day 8, and declined by Day 12.

In the present study we also observed that leukaemic sera from which viral particles were removed by centrifugation could also block the cytotoxic reaction of Rat ILR-3 and goat anti-$gs_3$. That virus was present in the sera of the animals bearing leukaemia was established by observing viral particles by electron microscopy in the pelleted material of leukaemic sera subjected to high speed centrifugation. Virus was first detected in sera from animals who received leukaemic cells 8 days previously, and by 12 days after passage of the leukaemia viral particles had markedly increased. Thus, the presence of the group specific internal virion protein, p30, in Moloney leukaemic sera and on leukaemic cells reaches a maximum level by Day 7–8 and declines, and thereafter intact virus particles are detectable and continue to increase in number. These time course relationships are consistent with assembly and production of new virus at the cell surface and release into the circulation. It is also of interest that the maximum levels of p30 are seen in the sera and on leukaemic cells at a time when maximum rate of growth of the leukaemia occurs.

Elimination of viral particles from leukaemic sera by centrifugation had no depressive effect on the ability of the sera to absorb the cytotoxic activity of Rat ILR-3 against MSC targets. This suggests that the internal virion protein, p30, present in both spun and unspun preparations was the major substance responsible for block in that system. This is compatible with our previous observations that for a given number of MSC targets the amount or accessibility of p30 for cytotoxic reactions was greater than that observed for M targets (Epstein and Knight, 1975). We showed that a given amount of purified p30 blocked the reaction of Rat ILR-3 against M targets more completely than that against MSC targets, where presumably there was more antigen to block.

In contrast, elimination of viral particles from leukaemic sera did have a depressive effect on the ability of the virus free sera to absorb the cytotoxicity of Rat ILR-3 for M targets. Virus free sera were still capable of blocking (Fig. 3) but to a lesser degree than with intact virus. This occurred for 2 reasons. First there was a depression in the level of p30 in Day 12 serum as compared with Day 7 or 8, and small changes in p30 levels would have significant effects on blocking ability in a system utilizing target cells such as the M cells which had relatively less p30 present or accessible then MSC cells. Second, antigens associated with the intact virion, possibly VEA, have been shown to be involved in the reaction of Rat ILR-3 with M cells, since intact MSV-M as well as purified VEA preparations can absorb some of the cytotoxic activity.

The discovery and identification of p30 as a component of leukaemic serum are compatible with several previous observations in the literature. First, Stuck *et al.* (1964) described the presence of soluble antigen in the plasma of mice with Rauscher virus induced leukaemia, distinct from infective virus particles, and with the same specificity as the cellular antigens of this leukaemia. Similar material was found in the plasma of mice with leukaemia induced by Moloney or Friend virus. Geering, Old and Boyse (1966) have also described a group specific soluble antigen demonstrable by immuno-precipitation, which was shared by both Gross and Friend, Moloney or Rauscher induced leukaemias. Our results are thus compatible with their observations and in agreement with Ferrer's recent suggestion that p30 is the molecular equivalent of GCSA (b), a group specific antigen on the surface of Gross virus induced lymphoma cells. We have extended these observations to identify p30 both on the surface of Moloney virus infected or transformed cells and in the sera of mice bearing Moloney leukaemia.

The nature of the material which we found in normal Balb/c sera and which partially blocks the cytotoxic reaction of Rat ILR-3 and the goat anti-$gs_3$ is still uncertain. Moloney found no evidence of horizontal transmission of Moloney leukaemia to control mice housed even in the same cage (Moloney, 1962) and the control mice used in the present study were kept even further away in separate cages. Further, we found no electron microscopic evidence of virus in pelleted material obtained from normal serum subjected to high speed centrifugation, thus ruling out the possibility of active viral infection in the control mice. In addition, the stability to storage at $-20°C$ of the material in control sera was less than that of the p30 in leukaemic serum. It is of interest that Abelev and Elgort (1970), using an indirect immunoautoradiographic technique, found trace quantities of group specific internal virion antigens of murine leukaemia viruses in the sera of normal mice of one strain of low leukaemia incidence (*i.e.* C57Bl/6), but not in the serum of Balb/c mice, also considered to be a low leukaemia incidence strain. Further characterization of the material found in normal Balb/c sera in the present study would therefore be of interest.

Recently, Shellam and Knight (1974) demonstrated that serum from rats bearing progressively growing Gross virus induced syngeneic lymphomata will block T lymphocyte cytotoxicity for tumour targets. p30 is also implicated in this system, as these same authors have shown that purified preparations of the protein can partially block T cell cytotoxicity and completely block syngeneic antibody mediated cytotoxicity (Knight *et al.*, 1975).

Thus, in this and in a previous paper (Epstein and Knight, 1975) we have defined a system in which a group specific internal virion protein is associated with and serves as a cytotoxic target on the surface of Moloney virus infected or transformed cells, and is present in the sera of mice bearing Moloney leukaemia. Whether p30 serves to abrogate host defence mechanisms *in vivo* as well as *in vitro* and in what form it exists in the sera, *i.e.* as free antigenic determinants or antigen–antibody complexes remains to be established.

The authors wish to thank Mr Roy Mahoney for preparing the electron micrographs.

### REFERENCES

Abelev, G. I. & Elgort, D. (1970) Group-specific Antigen of Murine Leukemia Viruses in Mice of Low Leukemic Strains. *Int. J. Cancer*, **6**, 145.

Aoki, T., Herberman, R. B., Johnson, P. A., Liu, M., & Sturm, M. M. (1972) Wild-type Gross Leukemia Virus Classification of Soluble Antigens. *J. Virol.*, **10**, 1208.

Epstein, L. B. & Knight, R. A. (1975) Studies on Mouse Moloney Virus Induced Tumours: I. The Detection of p30 as a Cytotoxic Target on Murine Moloney Leukaemic Spleen cells, and on an *in vitro* Moloney Sarcoma Line by Antibody Mediated Cytotoxicity. *Br. J. Cancer*, **31**, 499.

GEERING, G., OLD, L. J. & BOYSE, E. A. (1966) Antigens of Leukemias Induced by Naturally Occurring Murine Leukemia Virus: their Relation to the Antigens of Gross Virus and other Murine Leukemia Viruses. *J. exp. Med.*, **124**, 753.

GORCZYNSKI, R. M. & KNIGHT, R. A. (1975) Cell-mediated immunity to Moloney Sarcoma Virus in Mice II. Analysis of Antigenic Specificities Involved in T Lymphocyte-mediated *in vivo* Rejection of Murine Sarcoma Virus-induced Tumours. *Eur. J. Immunol.* In the press.

GORCZYNSKI, R. M., KILBURN, D. G., KNIGHT, R. A. *et al.* (1975) Nonspecific and Specific Immunosuppression in Tumour-bearing Mice by Soluble Immune Complexes. *Nature, Lond.*, .

HELLSTRÖM, I. & HELLSTRÖM, K. E. (1969) Studies on Cell-mediated Immunity and its Serum-mediated Inhibition in Moloney-virus-induced Mouse Sarcomas. *Int. J. Cancer*, **4**, 587.

HIRSCH, J. & FEDORKO, M. E. (1968) Ultrastructure of Human Leukocytes after Simultaneous Fixation with Glutaraldehyde and Osium Tetroxide and " Postfixation " in Uranyl Acetate. *J. cell Biol.*, **38**, 615.

KNIGHT, R. A., MITCHISON, N. A. & SHELLAM, G. R. (1975) Studies on a Gross Virus-induced Lymphoma on the Rat. II. The Role of Cell Membrane Associated and Serum p30 Antigen in the Antibody and Cell-mediated Response. *Int. J. Cancer*. In the press.

MOLONEY, J. B. (1962) The Murine Leukemias. *Fedn Proc.*, **21**, 19.

SHELLAM, G. R. & KNIGHT, R. A. (1974) Antigenic Inhibition of Cell-mediated Cytotoxicity Against Tumour Cells. *Nature, Lond.*, **252**, 330.

SJÖGREN, H. O., HELLSTRÖM, I., BANSAL, S. C. & HELLSTROM, K. E. (1971) Suggestive Evidence that the Blocking Antibodies of Tumor-bearing Individuals may be Antigen–Antibody complexes. *Proc. natn. Acad. Sci, U.S.A.*, **68**, 1372.

STUCK, B., OLD, L. J. & BOYSE, E. A. (1964) Occurence of Soluble Antigen in the Plasma of Mice with Virus-induced Leukemia. *Proc. natn. Acad. Sci, U.S.A.*, **52**, 950.